# The Role of ATRX in the Alternative Lengthening of Telomeres (ALT) Phenotype

**DOI:** 10.3390/genes7090066

**Published:** 2016-09-19

**Authors:** João P. Amorim, Gustavo Santos, João Vinagre, Paula Soares

**Affiliations:** 1 Instituto de Investigação e Inovação em Saúde (i3S), Universidade do Porto, Porto 4200-135, Portugal; jamorim@ipatimup.pt (J.P.A.); gsantos@ipatimup.pt (G.S.); jvinagre@ipatimup.pt (J.V.); 2 Instituto de Patologia e Imunologia Molecular da Universidade do Porto (Ipatimup), Porto 4200-135, Portugal; 3 Instituto de Ciências Biomédicas Abel Salazar da Universidade do Porto, Porto 4050-313, Portugal; 4 Departamento de Patologia e Oncologia, Faculdade de Medicina da Universidade do Porto, Porto 4200-139, Portugal

**Keywords:** ATRX, ALT, telomeres, telomerase

## Abstract

Telomeres are responsible for protecting chromosome ends in order to prevent the loss of coding DNA. Their maintenance is required for achieving immortality by neoplastic cells and can occur by upregulation of the telomerase enzyme or through a homologous recombination-associated process, the alternative lengthening of telomeres (ALT). The precise mechanisms that govern the activation of ALT or telomerase in tumor cells are not fully understood, although cellular origin may favor one of the other mechanisms that have been found thus far in mutual exclusivity. Specific mutational events influence ALT activation and maintenance: a unifying frequent feature of tumors that acquire this phenotype are the recurrent mutations of the Alpha Thalassemia/Mental Retardation Syndrome X-Linked (*ATRX*) or Death-Domain Associated Protein (*DAXX*) genes. This review summarizes the established criteria about this phenotype: its prevalence, theoretical molecular mechanisms and relation with ATRX, DAXX and other proteins (directly or indirectly interacting and resulting in the ALT phenotype).

## 1. Introduction

Telomeres are DNA–protein structures that protect chromosome ends, and consist of arrays containing guanine-rich (G-rich) repeats (TTAGGG)_n_ in vertebrates. These structures prevent the eventual loss of coding DNA due to the end replication problem, a limitation that causes telomere shortening within each cell division and leading, eventually, to cellular senescence or apoptosis [1]. In most tumors, the maintenance of telomeres, critical for genomic stability and immortality, is achieved by upregulation of the enzyme telomerase, which adds repeats to these structures, thus allowing novel cell divisions [2]. This enzyme is normally expressed during development and is composed of a reverse transcriptase catalytic subunit (telomerase reverse transcriptase (TERT)) and a RNA template (telomerase RNA component (TERC)) subunit.

Human adult somatic cells usually repress telomerase expression, although the enzyme continues to be expressed in proliferative cells (germ cells and tissue stem cells). As previously shown, most neoplastic cells de-repress their expression to support immortalization and tumor progression [2]. However, approximately 10% to 15% of cancers achieve immortalization via a telomerase-independent mechanism of telomere lengthening, the alternative lengthening of telomeres (ALT) [3], which was first observed in a telomerase-null mutant yeast, even-short telomeres 1 (EST1) [4]. These cells depend on a homologous recombination (HR) DNA-repair mechanism to maintain telomere length [4,5] and are characterized by the presence of: heterogeneous telomere lengths; the observation of ALT-associated promyelocytic leukemia bodies (APBs) that differ from common promyelocytic leukemia (PML) bodies found in other cell types by the inclusion of telomeric DNA and numerous specific recombination factors [6]; and telomere recombination with the presence of extrachromosomal (linear and circular) telomeric repeats (ECTRs) [7]. These features are consistent with hyperactive HR at telomeres, a feature not observed elsewhere in the genome so frequently, and the extrachromosomal telomeric DNA has been proposed to serve as a template for the extension of telomeres [8].

Cancers that have a mesenchymal origin are reported to activate ALT more frequently, while epithelial cancers rely more frequently on telomerase reactivation/re-expression [9,10]. As mesenchymal stem cells are known to express minimal or no detectable amounts of telomerase [11], this may predispose cells from this lineage to depend on ALT activation more frequently. Genome mapping by somatic cell hybridization technique has already demonstrated that ALT activation occurred through the loss of one or more repressor molecules present in normal somatic telomerase positive cells [12]. More recently, with the appearance of mass genome sequencing techniques, inactivating mutations were identified in the alpha thalassemia/mental retardation syndrome X-linked (*ATRX*), death-domain associated protein (*DAXX*) and Histone 3.3 complex in ALT-positive tumor cells, mainly in pancreatic endocrine tumors [13], glioblastoma multiforme, oligodendrogliomas [14] and others. Moreover, it has been shown that a crucial step in activating ALT and the consequent appearance of the ALT phenotype can in part be a consequence of *ATRX* or *DAXX* inactivating mutations [15,16].

ATRX is a chromatin remodeling protein that presents a SWI/SNF2-type ATPase/helicase motif but also has a plant homeodomain-like zinc finger [17,18]. It can be considered an ATP-driven DNA translocase belonging to the SWI/SNF family of chromatin landscapers [17]. DAXX is a highly conserved protein associated with both nuclear and cytoplasmatic events during apoptosis. Both proteins localize mainly in the nucleus and are associated with PML nuclear bodies and other subnuclear domains [19]. It has been demonstrated that DAXX functions as a histone H3.3 chaperone and, together with ATRX in a replication-independent chromatin assembly pathway, facilitates the incorporation of the histone variant H3.3 into telomeric and pericentromeric chromatin [20]. Recent studies have demonstrated that G-rich repeats can lead to the formation of non-B DNA structures, such as the G-quadruplexes DNA (G4-DNA) structures [21,22]. These structures represent an obstacle to multiple nuclear processes mainly due to their capacity of inducing replication fork stalling, leading to replicative stress and ultimately DNA damage [23]. It has also been shown that the ATRX protein can bind these G4-DNA structures in vitro [24] and that *ATRX*-null cells have difficulty in resolving them [25]. This restricting replicative stress function may help explain the tumor-suppressive role recently ascribed to this protein. ATRX seems to interact with many other proteins such as components of the MRN (Mre11, Rad50 and Nbs1) complex [26] that play key roles in genomic stability and replication, including the restart of stalled replication forks and double strand breaks repair (via HR or non-homologous end joining (NHEJ)) [27].

Here, we review the established notions and concepts about the ALT phenotype: its prevalence in cancer, the known molecular mechanisms and relation with ATRX, DAXX and other proteins that interact directly or indirectly with ALT.

## 2. Prevalence of ALT in Human Cancer Subtypes

Over the last years, many reports have been published addressing the prevalence of the ALT phenotype in several human cancer subtypes. To determine the presence of ALT, many techniques are used, either alone or in combination. Of all the methods available, telomere-specific fluorescent in situ hybridization (Tel-FISH) is one of the most commonly used techniques and, in the presence of cells with ALT, it detects telomeres that exhibit a variable and unbalanced size with the characteristic very bright intranuclear foci of telomeric sequence aggregates. It is also possible to combine Tel-FISH with the immunofluorescence of the PML protein that are present in the APBs and therefore this co-localization of PML and Tel-FISH aids in the identification of ALT presence. Other methods focus on the quantification of the ALT-associated molecules, such as the C-circles (and G-circles) detection. In some models, such as in the pancreatic endocrine tumors, absence of ATRX nuclear expression by immunohistochemistry can be used as surrogate marker of ALT phenotype; however, it should not be used without other confirmatory techniques [14]. The previous techniques represent an evolution towards the initial methods, such as the telomere restriction fragment assay, that were used mainly for determining telomere length. In Table 1, we present a comprehensive review of this phenotype prevalence.

Besides ALT, which is present in some tumor models (Table 1), other mechanisms for telomere maintenance are also available, such as *TERT* promoter mutations, as is the case for central nervous system (CNS) tumors. In CNS tumors, mainly in glioblastomas, where *TERT* promoter and *ATRX* mutations are mutually exclusive, it appears that both genetic mechanisms can confer similar advantages [56]. ALT is more frequently detected in secondary glioblastomas that are recognized to arise from lower grade precursor lesions in which *ATRX* mutations occur more frequently [57]. When addressing primary glioblastomas, which are believed to arise de novo, ALT is less frequently observed and *TERT* promoter mutations start to become very frequent; they are the main mechanism for telomere maintenance and associated to a worse prognosis [57,58]. Still, *TERT* promoter mutations are rare in pediatric tumors of the CNS [59]. In the pediatric tumors, another mechanism has a fundamental role in upregulation of telomerase expression, the hypermethylation of the *TERT* promoter [60]. This hypermethylation represents, so far, an unknown mechanism; one hypothesis is that these methylated areas could prevent the binding of a repressor protein and, therefore, telomerase to be expressed. These findings are consistent with the fact that the cells from which pediatric CNS tumors are thought to originate still have active telomerase, not requiring its reactivation through *TERT* promoter mutations. Another mechanism of telomerase reactivation recently unveiled in pediatric tumors (in this case from the sympathetic nervous system—neuroblastomas), were the *TERT* rearrangements. These were responsible for juxtaposing telomerase next to strong enhancer elements and this induced epigenetic remodeling of the *TERT* locus, resulting in a higher expression of the protein [61].

## 3. ALT Mechanisms

### 3.1. Alternative Lengthening of Telomeres

As previously described, in 10% to 15% of human cancers where no telomerase activity is detected, telomere length is assured by an HR mechanism. The first observation of this phenomenon was performed in a telomerase knock-out model in yeast, where it was discovered that these cells maintained telomere length without telomerase activity, but, if a double knock-out for *RAD52* was performed, the cells did not survive [4]. This sheds light how the ALT mechanism could operate, since this protein is critical for double strand break (DSB) repair by an HR mechanism. Moreover, studies using exogenous DNA integration into telomeric regions with tagged DNA were able to demonstrate that, in ALT cells, telomeric DNA is copied to other telomeres [5].

**Figure 1:**
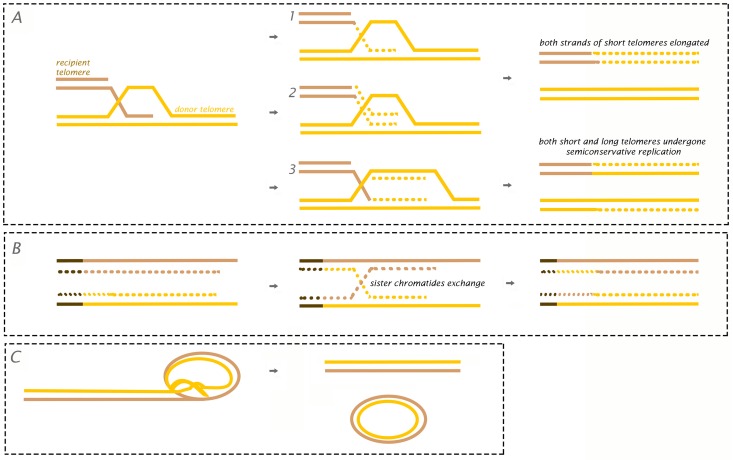
Theoretical models for ALT mechanism: (**A**) Break-induced replication loss from the donor telomere (1 and 2 without loss from the donor telomere). In Model 3, there is a unidirectional replication fork with semi-conservative replication of the telomeres; (**B**) Unequal telomeric sister chromatid exchange (T-SCE) model; (**C**) Rolling circle and t-circle formation after t-loop resolution providing a linear double strand break for subsequent HR-mediated activation into the homologous templates. Adapted from [62].

Currently, various proposed models describe how telomeres are maintained and extended without telomerase enzyme activity (Figure 1) [62]. The first (Figure 1A) represents a model based in break-induced repair (BIR), a mechanism where DNA is synthetized away from a break site using an HR donor template that, in this case, is telomeric DNA. Models 1 and 2 in Figure 1A differ in the timing of the lagging strand and, in both, there is loss of material in recipient telomeres. Model 3 represents a unidirectional replication fork that will require a Holliday junction in the end of the process and, in this case, both donor and recipient telomeres will be semi-conserved [63]. Unequal telomeric sister chromatid exchange (T-SCE) has also been suggested as a mechanism for ALT (Figure 1B). This is supported by the fact that T-SCE is elevated in ALT^+^ cells as measured by chromosome orientation fluorescence in situ hybridization (CO-FISH) [64,65]. Finally, the recurrent presence of ECTRs in ALT cells suggests that these molecules can be used as templates for HR-driven telomere elongation or simply undergo rolling-circle replication (Figure 1C) [62].

### 3.2. ATRX and DAXX Proteins Role in ALT

*ATRX* gene is localized in chromosome Xq21.1 and it encompasses 37 exons [66,67]. This gene encodes a chromatin remodeler which is a 280 kDa protein that includes an unusual N-terminal plant homeodomain (PHD) designated the ATRX-DNMT3-DNMT3L (ADD) domain [68,69,70]. At the C terminus it presents seven helicase subdomains that confer ATPase activity and identify ATRX as a SNF2 family member of chromatin-associated proteins [71]. As previously mentioned, it is known that ATRX, in collaboration with its partner DAXX, functions as a complex for the deposition of the histone variant H3.3 into telomeric and pericentromeric chromatin. DAXX is a highly specific histone chaperone that is able to discriminate H3.3 from the other major histone variants, whereas ATRX is involved in targeting DAXX to repetitive sequences enhancing histone deposition [72,73].

**Figure 2:**
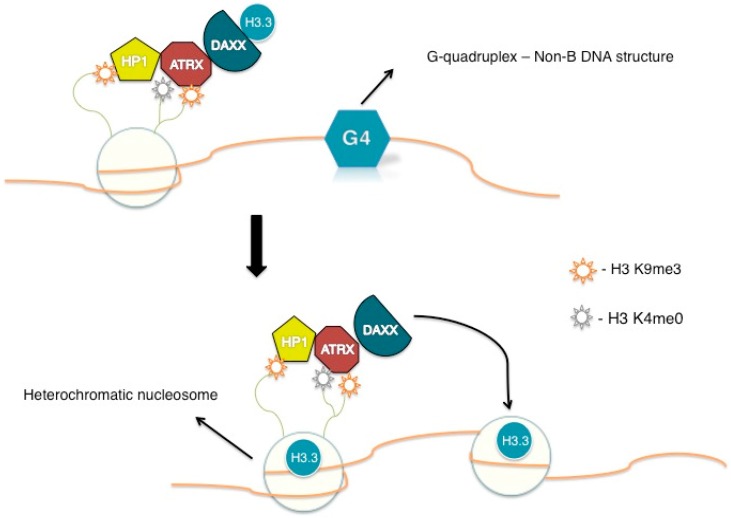
A proposed theoretical mechanism for Alpha Thalassemia/Mental Retardation Syndrome X-Linked/Death-Domain Associated Protein (ATRX/DAXX) chromatin landscaping: ATRX binds to histone H3 at heterochromatin through interaction of its ATRX-DNMT3-DNMT3L (ADD) domain with an H3 N-terminal tail, trimethylated at Lys9 (H3K9me3) and unmodified at Lys4 (H3K4me0). HP1 also recognizes H3K9me3, boosting this recruitment. Once in its target site, ATRX, in combination with DAXX, facilitates the deposition of the histone variant H3.3 [80]. The deposition of H3.3 leads to changes in chromatin that prevent the formation of G4-DNA structures. HP1: heterochromatin protein 1.

Immunofluorescence studies have demonstrated that ATRX has a preference for binding within PML bodies and also to repetitive heterochromatic regions such as ribossomal DNA (rDNA), telomeric and pericentric DNA repeats [17,74,75]. The localization of ATRX to heterochromatin involves the interaction with the heterochromatin protein 1 (HP1) [76,77,78]. In addition, targeting of ATRX to heterochromatin is dependent on its interaction with histone H3 trimethylated Lys9 (H3K9m3) and unmodified Lys4 (H3 K4m0) [79]. One hypothesis is that HP1 might serve as a protein scaffold, facilitating the recruitment of ATRX to the heterochromatin through binding of H3K9me3 via its N-terminal chromodomains [79,80] (Figure 2). Still, this recruitment seems to be of extreme complexity and rely on multiple interactions between effector proteins and chromatin.

It has been reported that heterochromatic regions are prone to DNA damage in the absence of the ATRX protein [15,25]. This could mean that it plays a role in one of the two DNA DSB repair mechanisms (NHEJ in post mitotic cells; HR in proliferative ones). However, the same authors that stated the previous affirmations, exposed ATRX-KO cells to irradiation and concluded that the protein is not required for repair of induced DSBs and, therefore, its role might be protecting cells from replicative stress instead of repairing them [25,81]. Interestingly, and in contrast with the latter, a more recent approach to this question by Koschmann et al. brought new insights regarding the role of ATRX in DNA-repair pathways. The authors used an animal model of *ATRX*-deficient glioblastoma to uncover the impact of this protein loss in tumor proliferation and loss of genetic stability. They found out that the reduction of ATRX levels impaired NHEJ pathway, as co-transfection with a DNA-damaged GFP designed to be restored by NHEJ and ATRX silencing reduced NHEJ function by 50% [82]. These findings suggest that a relative increase in HR in relation to NHEJ in *ATRX*-deficient gliomas may be supportive of ALT. In addition, these data are explicative of the genetic instability, and altered DNA damage response in ALT cell lines [15,81,82], besides providing a molecular target that can be explored to design new targeted therapies regarding ALT-positive gliomas.

Telomeres composition of repetitive DNA with an high content of guanine and cytosine residues leads to the formation of secondary DNA structures, the previously mentioned G4-DNA structures [22]. These are known to form obstacles (like bulky DNA adducts) to multiple nuclear processes including DNA replication and transcription. A large proportion of ATRX target sites are predicted to adopt non-B DNA structures like these G4-DNA structures [24], leading to the concept that this protein might be responsible for aiding in replication in the presence of G4-DNA structures or by preventing their formation, and therefore facilitating replication at telomeres. A recent study suggests that ATRX does not appear to possess G4-DNA unwinding activity and that it might overcome these impediments indirectly, perhaps by favoring the histone H3.3 deposition to maintain DNA in a B-form conformation (Figure 2) or by promoting a fork bypass via template switching [26,83]. Furthermore, a potential consequence of loss of ATRX function is an increased frequency of these G-quadruplexes, causing DNA damage [84]. Thus, it is likely that the presence of such structures in telomeric DNA favors the ALT phenotype by presenting a barrier to the replication fork, causing fork stalling, collapse and subsequent restart by the homologous recombination DNA-repair mechanism [26].

Depletion of proteins or protein-complexes that are involved in HR and/or are required for replication fork restart like MRN complex, SMC5/6 complex, MUS81 or the Bloom syndrome protein (BLM) helicase (some of which we will discuss further in this review) suppress telomeric recombination in ALT cells [85,86]. Particularly, the MRN complex functions includes DSB repair (via HR or NHEJ) and the restart of stalled replication forks [87] and, like ATRX, it co-localizes to telomeres during the S- and G2-phases of the cell cycle [88]. In addition, G-quadruplexes were found to be a preferred substrate for the MRX (MRN yeast homologue) complex-component MRE11 [89]. The association of MRN complex with PML bodies (constituting the APBs) seems to be a requirement of ALT activity and likely constitutes the sites of telomeric recombination [5]. These data show that the proteins in the MRN complex might have an important role in the ALT mechanism as they interact with ATRX, PML bodies and telomeres.

Recently, it was demonstrated that ectopic expression of ATRX in an ALT-positive cell line significantly reduced replication fork stalling, likely limiting the substrate for HR and downregulating ALT [26]. In addition, the same authors had already revealed that ATRX interacts with endogenous MRN complex components in a direct manner during DNA replication, suggesting that this interaction is likely to play a direct role in facilitating this process [83]. Moreover, the re-expression of ATRX in ALT cell lines resulted in a dramatic redistribution of the MRN complex away from telomeric DNA and PML nuclear bodies [26]. Overexpression of a PML nuclear body component, Sp100, also showed sequestration of MRN components away from APBs leading to suppression of ALT [85]. These data suggest that ATRX expression in a cell with the ALT phenotype may limit ALT by redistributing MRN away from sites of telomeric recombination. The fact that G4-DNA are a substrate for MRE11 raises the possibility that MRN directly cleaves these structures triggering DBS formation and HR [26].

All available data have led us to propose that the lack of ATRX is linked to ALT by two main mechanisms, which can act independently or not: (i) without this protein there is an increase in stalled replication forks due to G4-DNA structures that promote HR; and (ii) the redistribution of the MRN complex to APBs may also promote this DNA-repair mechanism.

## 4. ALT-Associated Proteins

Telomere maintenance is regulated by complex interactions of multiple proteins, transcription factors, signaling pathways and epigenetic events. Specific alterations in these regulation mechanisms in tumors might facilitate ALT activation and/or maintenance through recombination-mediated telomere maintenance. This stresses the importance of understanding the biological interactions between proteins in this context, in order to better comprehend the ALT phenotype. We have previously discussed, in this review, the most important known proteins in ALT (ATRX, DAXX, H3.3 and direct partners). Even so, there are numerous other proteins and complexes that should be addressed when studying the ALT phenotype as presented in Table 2.

Mutant proteins that might be permissive for ALT are those that affect telomerase regulation. Recent work suggests that the ALT phenotype emerges when telomerase expression is inhibited and that ALT cells actively repress telomerase expression through complex signaling networks [90]. *C-MYC*, e.g., activates telomerase through induction of *TERT* expression. Its inhibitor *TCEAL7* is upregulated in ALT cell lines, suggesting that a balance between these factors might determine whether there is telomerase expression or ALT activation [91].

Although it is important to note that proteins like p53 or multiple endocrine neoplasia type 1 (MEN1) which, when mutated, might be permissive for ALT, we will now focus on molecules that are more directly related to the molecular hallmarks of ALT. These are mostly related to the maintenance of this phenotype by telomere recombination. Depletion of proteins or complexes that are involved in HR and are required for replication fork restart like RAD51, RAD52, the MRN complex, the SMC5/6 complex, MUS81 or the Werner syndrome RecQ like helicase (WRN) helicase, indeed suppress telomeric recombination in ALT cells, resulting in telomere shortening, loss of APBs or alterations in other ALT-specific characteristics [92].

As mentioned above, ALT cell lines and tumors contain APBs, in which PML protein co-localizes with telomeric DNA and the telomeric repeat-binding factor 1 and 2 (TRF1 and TRF2) proteins [6,93]. These structures also contain proteins that are important in DNA processing, namely in recombination—RAD51 and RAD52 [93]. Single-ended DSBs can occur at telomeres or at broken replication forks and can be repaired by HR through a single-ended invasion process known as break-induced replication (BIR) [94]. Most BIR events are dependent on RAD51 [95]. RAD51 knockdown in ALT cell lines results in telomere dysfunction and apoptosis [96], although this effect might be independent of ALT mechanisms, as different authors observed that RAD51 depletion was not found to have any effect on ALT activity [97,98]. In many organisms, RAD52 is a key protein involved in the HR pathway. Interestingly, although this protein itself did not demonstrate recombination mediator activity in reconstituted biochemical assays, it appears to mediate RAD51 function in the homologous recombination machinery [99,100]. Moreover, it was discovered that, in a telomerase-null mutant yeast (EST1^−^), telomere length was maintained. However, if a *RAD52* knock-out was performed, cells became unviable [4].

The MRN complex functions in detection, signaling and resolution of DSBs in the genome and its suppression by over-expression of SP100 (the PML nuclear bodies major component) or inhibition of its component NBS1 results in shortened telomeres, loss of APBs and impaired formation of extrachrossomal telomeric repeats, suggesting impaired ALT [85,101].

The SMC5/6 complex promotes homologous recombination-mediated repair of DNA DSBs. It localizes to APBs and is required for the inclusion of telomeric DNA in these bodies. Its depletion also results in shortened telomeres and cellular senescence in ALT cells [97]. Moreover, the MMS21 SUMO ligase within the SMC5/6 complex stimulates the sumoylation of multiple subunits of the shelterin complex and this modification is required for APB formation [97].

Shelterin is a protein complex formed by six telomere-specific proteins (TRF1, TRF2, protection of telomeres 1 (POT1), TRF1-interacting nuclear protein (TIN1), tripeptidyl peptidase 1 (TPP1) and repressor / activator protein 1 (RAP1)). It associates with the arrays of TTAGGG repeats and protects chromosome ends functioning as a telomere cap, as it distinguishes telomeres from sites of DNA damage. Without the protective activity of shelterin, telomeres are no longer hidden from the DNA damage surveillance and DNA repair pathways inappropriately process chromosome ends [102]. It has been shown that TRF2-depleted U-2 OS and SUSM1 clones display shorter telomeres and decreased telomeric signals, describing an effect of the shelterin component TRF2 knockdown on telomeric DNA in ALT cells [103]. However, no mutations in TRF2 have been identified to cause ALT. Furthermore, variant telomeric repeats which do not bind TRF2 have been found in ALT cells [104].

BLM and WRN are helicases that unwind DNA, allowing MRN and SMC5/6 complexes to access telomeric DNA, so HR can occur. WRN has a specific domain with exonuclease activity and its silencing, (RNAi)-mediated depletion, was capable of inhibiting ALT [105]. Specifically, WRN continuous knockdown managed to reduce telomere length and inhibit APBs formation, suggesting a role in telomere maintenance and APBs formation [105]. BLM helicase seems to interact with TRF2 (shelterin component) protein, and its overexpression led to rapid, ALT cell-specific increases in telomeric DNA synthesis [106]. In addition, the majority of BLM foci co-localized with telomere foci and this behavior was restricted to ALT cells [106]. Other proteins associated with ALT may not be directly related to telomere recombination to maintain telomere length per se. Nonetheless, they may have other functions as cell cycle control, telomere damage signaling or structural integrity. These include X-ray repair cross-complementing protein 3 (XRCC3), MUS81, replication protein A (RPA), Ku70/80 or flap endonuclease 1 (FEN1).

The ASF1 (Anti-Silencing Factor 1) paralogs ASF1a and ASF1b are histone chaperones that are responsible for assisting the transfer of H3.1-H4 histone dimers to chromatin assembly factor 1 (CAF-1) or H3.3-H4 histone dimers to histone regulator A (HIRA), for nucleosome assembly [107,108]. Co-depletion of both paralogs in human cells induced all hallmarks of ALT. These included the formation of ALT associated PML bodies (APBs), extra-chromosomal telomeric DNA species, an elevated frequency of T-SCE events and inter-telomeric exchange of an integrated tag. The induction of ALT characteristics in this setting also led to the suppression of telomerase [109].

The SWI/SNF related, matrix-associated, actin-dependent, regulator of chromatin, subfamily A-like 1 (SMARCAL1) DNA translocase is one of several related enzymes that are recruited to stalled replication forks to promote repair and restart replication [71,110,111]. Pooleet al. have shown that SMARCAL1-deficient cells accumulate telomere-associated DNA damage and have elevated levels of extrachromosomal telomere DNA (C-circles). Although these telomere phenotypes are often found in tumor cells using the ALT pathway for telomere elongation, SMARCAL1 deficiency does not yield other ALT-associated characteristics such as elevated telomere recombination [112].

XRCC3 is a protein component of the homologous recombination machinery, specifically involved in resolving Holliday junctions and its knockdown reduces ECTRs in ALT cells [101].

MUS81 is an endonuclease implicated in DNA replication fidelity as it processes replication intermediates. Its loss induces growth arrest and inhibits TSCE in ALT cells, although telomere shortening is not seen. The loss of this protein does not seem to affect telomerase-positive cells, suggesting that its telomeric functions are specific to the ALT phenotype [86].

RPA binds to single-stranded DNA (ssDNA) to stabilize intermediate structures in replication, repair and recombination. Its knock down induced growth arrest and accumulation of telomeric ssDNA in ALT cells [113].

The Ku70/80 heterodimer binds to broken DNA ends to facilitate NHEJ repair pathway. Its loss inhibits proliferation of ALT cells and reduces ECTRs, although it does not provoke telomere shortening [114].

FEN1 is a protein that processes DNA replication intermediates. Its knockdown in ALT cells increases the telomeric DNA damage response, suggesting that it is required for telomere stability in these cells [115].

These proteins are, thus, not required for telomere length maintenance, as their loss does not provoke telomere shortening. Even so, they seem to be related to ALT telomere structure preservation.

p53 is a DNA binding and signaling protein that functions as a transcription factor, activating or repressing a large number of target genes. It also plays an important role in cell cycle progression control and in facilitating DNA repair [116]. Mechanistically, *TP53* mutations alter HR processes and could be permissive to ALT through loss of recombination suppression. Mouse models have shown that *TP53* mutation disrupts cell cycle checkpoint control and DNA damage signaling [92,117]. In the absence of telomerase this enables tumor progression, likely through activation of ALT [92]. There are also clinical data suggesting that *TP53* mutations may be permissive for ALT in human tumors; e.g., mutations in this gene are associated with ALT in human gliomas of the brain and spinal cord, a tumor type with high incidence of ALT phenotype [118]. Nevertheless, it is important to have in mind that p53 has multiple tumor suppressor functions other than that which is related to telomere maintenance.

*MEN1* and phosphatase and tensin homologue (*PTEN*) are two tumor suppressor genes that are frequently mutated in pancreatic neuroendocrine tumors (pNETs). *MEN1* is somatically mutated in 22%–34% of sporadically occurring pNETs [119], whereas loss of chromosome arm 10q, where *PTEN* is located, occurs in 25% of pNETs [120,121]. The *MEN1* gene codes for the menin protein, a component of the MLL/SET1-like histone methyltransferase complex that regulates chromatin remodeling and functioning as transcription factor [119,122]. *PTEN* (phosphatase and tensin homolog) encodes a dual lipid and protein phosphatase upstream of the oncogenic PI-3-kinase-Akt-mammalian target of rapamycin (mTOR) pathway [123]. In tumors with loss of function mutations of *PTEN*, unrestrained activation of this pathway results in cell growth and proliferation [119]. A recent study suggests that 56% of pancreatic neuroendocrine tumors are ALT-positive [124]. It remains now to be determined if mutations in these tumor suppressor genes could be permissive for ALT, or if ATRX loss, frequently detected in pNETs, by itself is the only factor. Interestingly, in MEN1 syndrome associated pNETs ALT is less frequently detected and TERT promoter mutations are also detected but are mutually exclusive [125].

## 5. ATRX Loss Promotes ALT in Mesenchymal Cells

A recent study evaluated the effect of knocking down ATRX in fibroblasts and epithelial cells, to determine whether loss of this gene promoted the ALT mechanism [126]. They found that ATRX depletion in epithelial cells does not promote ALT activation. However, in fibroblasts, ATRX knockdown led to an increased ALT frequency [126].

Embryonic stem cells are known to have telomerase activity. However, mesenchymal stem cells express little or no detectable telomerase [127] and this may be the reason why these cells do not activate telomerase in a similar frequency to epithelial cells. This suggests that telomerase expression may be repressed by continuous chromatin-mediated repression in the mesenchymal lineage. Another possible explanation may be that epithelial cells express proteins that mesenchymal cells do not and one or more of those might have a role in helping to overcome G4-DNA structures and/or chromatinization.

Although ATRX loss promotes ALT, it seems it is not enough to activate this alternative mechanism, even in fibroblast cell lines. Knocking down ATRX in fibroblasts was capable of activating ALT in 10 out of 12 cultures [126]; one of their six controls activated ALT. This leads to the concept that loss of ATRX is not enough to activate ALT and the presence of ATRX is not enough to avoid ALT. These data suggest that there is a missing link in this mechanism, which can be other protein(s) or (epi-) genetic changes that can interact with ATRX.

## 6. ATRX Expression Suppresses ALT

It has been proposed that ATRX acts as a tumor suppressor. A study conducted in telomerase-negative U-2 OS ALT cells, which are also null for ATRX, revealed that re-expression of ATRX in these cells suppresses the ALT phenotype and, if that re-expression is switched off, it is acquired again. However, if G-quadruplexes are stabilized (using pyridostatin), ALT suppression by ATRX is prevented. This study also revealed that this suppression is dependent on the histone chaperone DAXX as re-expression of ATRX in ALT cells concomitant with DAXX KO was not capable of suppressing the ALT phenotype [26].

Another study, conducted in three different ALT mesenchymal cell lines that lack ATRX expression, showed that ATRX re-expression represses the ALT mechanism [126], even though the cells had to enter crisis before the ALT phenotype emerged. The explanation for this event, as mentioned above, might be the fact that ATRX facilitates replication and resolution of the G-quadruplex secondary structures, not allowing replication fork stalling, subsequent DSB formation and activation of the HR mechanism (the latter is a known hallmark of ALT). Another possible explanation is that ATRX helps in the formation of telomeric chromatin, not allowing the formation of DNA secondary structures like G-quadruplexes, therefore potentiating the normal mechanism of telomere replication, with no intervention by the HR-mediated repair of DNA.

Finally, it is pertinent to say that, as the results of a recent study show, loss of ATRX also suppresses resolution of cohered telomeres as, in the absence of this protein, the histone variant macroH2A1.1 binds to the poly(ADP-ribose) polymerase tankyrase 1, preventing it from localizing to telomeres and resolving cohesion (as cohesion resolution is the known function of this specific polymerase) [128]. The persistent cohesion that follows loss of ATRX thus promotes recombination between sister telomeres (telomere sister chromatid exchange (T-SCE)), a known ALT hallmark. These data suggest that this phenomenon indeed controls recombination in ALT cells and points to a role of ATRX in the ALT phenotype, in addition to its DAXX/H3.3 histone chaperone function. Even though this does not resolve the question of whether ATRX has an unmistakable role in activating ALT, the possibility is raised that the precondition of persistent telomere cohesion might favor activation of the ALT pathway over the upregulation of telomerase during tumorigenesis [128].

## 7. Future Perspectives

In this review, we aimed to bring a better comprehension of the influence of ATRX and related proteins in the ALT phenotype. In addition, we wanted to establish new connections between the numerous proteins implicated in this phenomenon. We finished by exposing the most recent facts about the relation of the ATRX gene mutations with the generation of ALT phenotype. Further studies are needed to determine the exact relation of the mentioned proteins with the ALT phenotype with the aim of creating a reliable model for its origin and maintenance. In addition, identification of molecules that might serve as specific targets for therapy in ALT-positive tumors is a crucial aim. Since ALT is not detected in normal cells, targeting ALT^+^ cancer cells would be of great interest. Koschmannet al. highlighted the possibility of using the detection of the *ATRX* mutation or ATRX loss by immunostaining as evidence of the presence of a treatment-responsive subtype of glioma that would encourage the use of DSB-inducing agents as targeted therapy. In addition, these data raised the possibility of using topoisomerase inhibitors to exploit the defected DNA repair in ATRX-deficient gliomas [82]. Other strategies could include the design of small molecules or inhibitors that target ALT cells, e.g., the inhibition of ATR protein kinase, a critical regulator of recombination, recruited by the Replication Protein A. This inhibition has been proven to disrupt ALT and trigger chromosome fragmentation and apoptosis in ALT cells [129]. Finally, as hypothetically suggested by Ramamoorthy and Smith , it is possible that, once the macroH2A1.1-binding domain of ATRX is defined, a small molecule can be designed to target this histone variant’s domain with the aim of leaving tankyrase 1 free to resolve telomere cohesion, thus providing a molecular target [128]. All of these strategies are highly selective for cancer cells that rely on ALT, and thus represent a potential targeted therapy for ALT-positive cancers.

## Figures and Tables

**Table 1:** Prevalence of the alternative lengthening of telomeres (ALT) phenotype in human cancer subtypes.

Location/Tumor Type		% ALT^+^^a^	Reference	Clinical Behavior/Observations
*Adrenal gland Peripheral nervous system*				
Phaeochromocytoma		3	[9]	-
Neuroblastoma		9	[9]	ALT positivity was associated with significantly reduced Overall Survival [28]
		59	[28]	
Ganglioneuroblastoma		14	[9]	-
Adrenocortical carcinoma		12	[29]	-
*Breast*				
Ductal carcinoma		2	[9]	-
		4	[30]	
Lobular carcinoma		4	[9]	-
Medullary carcinoma		2	[9]	-
*CNS*				
Astrocytoma		34	[31]	More often in lower grades [31] Significantly lower mean age at diagnosis [31]
Pilocytic astrocytoma (grade 1)		3	[9]	-
Diffuse astrocytoma (grade 2)		63	[9]	-
Anaplastic astrocytoma (grade 3; adult)		63	[9]	-
		89	[32]	
Adult low-grade astrocytomas (grade 2)		27	[33]	-
Pediatric high-grade astrocytomas (grade 3 and 4)		29	[33]	Significant association between *PDGFRA* amplification and ALT positivity in both adult and pediatric high-grade astrocytomas [33] ALT phenotype is highly associated with ATRX loss [33]
Adult high-grade astrocytomas (grade 3 and 4)		26	[33]	
Primary glioblastoma (grade 4)		18	[32]	ALT phenotype positively correlated with the presence of round cells microcysts, IDH1 mutant protein, ATRX protein loss, strong p53 expression and absence of *EGFR* amplification [32]
Secondary glioblastoma (grade 4)		57	[32]	
Glioblastoma (grade 4; adult)		25	[34]	ALT mechanism is associated with longer survival [34] Overall median survival was significantly longer in patients with GBM positive for both ALT and *IDH1* mutation [35]
		15	[35]	
		11	[9]	
Glioblastoma (grade 4; pediatric)		44	[9]	Significantly increased prevalence in pediatric GBM, compared with adult [9] Associated with the presence of mutant H3F3A/ATRX [16]
		38	[16]	
Oligodendroglioma		20	[9]	-
Medulloblastoma, anaplastic		18	[9]	-
Medulloblastoma, non anaplastic		3	[9]	-
Other embryonal tumors		10	[9]	-
Meningioma		2	[9]	-
Schwannoma		2	[9]	-
*Childhood brain tumors*	Primitive neuroectodermal tumors	12	[36]	ALT attenuated the poor outcome conferred by *TP53* mutations in specific pediatric brain tumors (glioma and choroid plexus carcinoma) [36]
	Choroid plexus carcinomas	23	[36]	
	High grade gliomas	22	[36]	
*Oesophagus*				
Adenocarcinoma		1	[9]	-
*Gallbladder*				
Adenocarcinoma		2	[9]	-
*Kidney*				
Clear cell carcinoma		1	[9]	-
Papillary carcinoma		1	[9]	-
Chromophobe carcinoma		9	[9]	-
Sarcomatoid carcinoma		7	[9]	-
*Liver*				
Hepatocellular carcinoma		7	[9]	-
Hepatocellular carcinoma with abrupt anaplasia		92	[37]	-
*Lung*				
Small cell carcinoma		2	[9]	-
Large cell carcinoma		3	[9]	-
*Neuroendocrine Neoplasms*				
Carcinoid tumor		6	[9]	-
Paraganglioma		13	[9]	-
Ovary and testis				
Embryonal carcinomas		24	[38]	-
*Ovary and Testis*				
Clear cell carcinoma		4	[9]	-
Endometrioid carcinoma		1	[9]	-
Nonseminomatous germ cell		15	[9]	-
*Mesothelium*				
Malignant Mesothelioma		4	[9]	-
Diffuse Malignant peritoneal mesothelioma		22	[39]	ALT was associated with a younger age at diagnosis [39]
*Skin*				
Malignant melanoma		7	[9]	-
*Sarcomas*				
Undifferentiated pleomorphic sarcoma		63	[9]	Loss of ATRX was highly associated with alternative lengthening of telomeres in sarcomas (all subtypes) [40]
		65	[40]	
Fibrosarcoma and variants		14	[9]	-
Myxofibrosarcoma (myxoid variant of malignant fibrous histiocytoma)		76	[40]	-
Malignant fibrous histiocytoma (Pleomorphic sarcoma)		77	[31]	ALT-positive status emerged as the only independent prognostic factor for mortality [41]
		33	[41]	
Leiomyosarcoma		53	[9]	ALT-positivity associated with the epithelioid/pleomorphic cell morphology, tumor necrosis, poor differentiation, high FNCLCC grade and more aggressive behavior [42]
		59	[42]	
		62	[31]	
Liposarcoma		24	[9]	Higher frequency in dedifferentiated stages [43] ALT associated with disease-specific mortality [43,44]
		33	[31]	
		25	[43]	
		18	[44]	
		24	[45]	
Pleomorphic Liposarcoma		80	[46]	-
Dedifferentiated liposarcoma		30	[46]	ALT is the most significant factor that predicted a short progression-free survival [46]
Myxoid or round cell liposarcoma		5	[46]	-
Angiosarcoma		11	[9]	-
		24	[47]	
Epithelioid sarcoma		33	[9]	-
Malignant peripheral nerve sheath tumor		0	[9]	-
		21	[40]	
		37	[48]	
Rhabdomyosarcoma		0	[9]	-
		6	[31]	
Embryonal rhabdomyosarcoma		13	[40]	-
Chondrosarcoma		100	[9]	-
Neurofibroma		11	[9]	-
Radiation-associated sarcoma		20	[40]	-
Osteosarcoma		63	[49]	Significantly lower age at diagnosis for the ALT^+^ osteosarcoma patients compared with the ALT^−^ osteosarcoma patients [31]
		47	[31]	
		66	[50]	
		80	[51]	
Synovial sarcoma		9	[31]	-
*Stomach*				
Gastric carcinoma		0	[9]	-
		38	[52]	
MSI-H gastric carcinoma		57	[52]	-
Non-MSI-H gastric carcinoma		19	[52]	-
Medullary carcinoma		28	[53] ^b^	ALT associated with a low MIB-1 proliferation index [53]
*Urinary bladder*				
Invasive urothelial carcinoma		1	[9]	-
Small cell carcinoma		23	[9]	-
*Uterus*				
Cervix, squamous carcinoma		2	[9]	-
Endometrium, serous carcinoma		7	[9]	-

^a^ Indicated ALT frequencies are for primary (not metastatic) tumors. ALT was not found in the following cancer subtypes: cholangiocarcinoma [9], breast (ductal carcinoma with lobular features, mucinous carcinoma and tubular carcinoma) [9], pediatric low-grade and pilocytic astrocytomas [9], pediatric ependymoma [54], colon adenocarcinoma [9], esophagus squamous and small cell carcinomas [9], hematopoietic neoplasms (Non-Hodgkin’s lymphoma, diffuse large B-cell; Non-Hodgkin’s lymphoma, other subtypes; Hodgkin’s lymphoma, nodular sclerosis; Hodgkin’s lymphoma, mixed cellularity; thymoma) [9], larynx squamous cell carcinoma [9], Lung (adenocarcinoma, squamous cell carcinoma, papillary carcinoma, bronchioloalveolar carcinoma, carcinoid tumor and other subtypes) [9], oral cavity squamous cell carcinoma [9], ovary serous and mucinous carcinoma [9], testicular seminoma [9], pancreatic adenocarcinoma [9], prostate adenocarcinoma and small cell carcinoma [9], salivary gland carcinoma [9], pleural mesothelioma [55], skin squamous and basal cell carcinomas [9], small intestine adenocarcinoma [9], soft tissues (gastrointestinal stromal tumor, Kaposi’s sarcoma, Ewing’s sarcoma, well-differentiated liposarcoma [46], clear cell sarcoma) [9], tendon sheath giant cell tumor [9], thyroid follicular and papillary carcinoma [9], urinary bladder (non-invasive urothelial carcinoma, non-invasive papillary urothelial carcinoma, squamous and sarcomatoid carcinoma) [9] and uterus (cervix, adenocarcinoma; endometrioid carcinoma; endometrium, mixed mesodermal tumor; endometrium, clear cell carcinoma) [9]; ^b^ 11/39 cases were ALT^+^; 16 telomerase reverse transcriptase (TERT)-positive cases (from the total 39) were not studied regarding the ALT phenotype. CNS: central nervous system; ATRX: alpha thalassemia/mental retardation syndrome X-linked; IDH1: isocitrate dehydrogenase 1; EGFR: epidermal growth factor receptor; GBM: glioblastoma.

**Table 2:** Proteins that, when depleted or overexpressed in ALT cells, produce the listed telomere phenotypes.

Protein(s)	Cell Lines	Depletion Result
RAD51	WI38-VA13/2RA	Telomere dysfunction (reduced telomere length) and apoptosis [96]
RAD52	EST1^−^ Yeast	Senescence phenotype [4]
MRN COMPLEX	IIICF/c spontaneously immortalized Li-Fraumeni syndrome fibroblast line	Shortened telomeres, loss of APBs and impaired formation of ECTRs [85]
SMC5/6 COMPLEX	U-2 OS	Shortened telomeres, disruption of APBs formation and cellular senescence [97]
WRN HELICASE	VA-13 and U-2 OS	Reduced telomere length and inhibition of APBs formation [105]
ASF1	IMR90, WI38 and HeLa	Induction of ALT hallmarks: Formation of ALT-associated PML bodies and ECTRs [109]
SMARCAL1	HeLa 1.3 and U-2 OS	Accumulation of telomere-associated DNA damage and circular ECTRs (C-circles) [112]
SHELTERIN (TRF2)	U-2 OS and SUSM1	Shorter telomeres and decreased telomeric signals [103]
XRCC3	GM-847, WI-Va13 and Saos2	Reduced ECTRs [101]
MUS81	GM847, U2OS and SAOS-2	Growth arrest and inhibition of telomere sister chromatid exchange; Telomere shortening is not seen [86]
RPA	GM-847 and U-2 OS	Growth arrest and accumulation of G-rich telomeric ssDNA [116]
KU70/80	CCL75.1	Inhibition of the proliferation of ALT cells; Reduced ECTRs; Telomere shortening not seen [114]
FEN1	U-2 OS	Telomere dysfunction; Increase of the telomeric DNA damage response [115]
BLM HELICASE (Overexpressed)	WI38–VA13/2RA and GM-847	Overexpression result: Increases in telomeric DNA synthesis dependent on BLM helicase activity [106]

APBs: ALT-associated promyelocytic leukemia bodies; ECTRs: extrachromosomal (linear and circular) telomeric repeats; PML: promyelocytic leukemia; ssDNA: single stranded DNA; ALT: alternative lengthening of telomeres.
